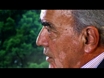# The 2009 Lindau Nobel Laureate Meeting: Werner Arber, Physiology or Medicine 1978

**DOI:** 10.3791/1571

**Published:** 2010-03-10

**Authors:** Werner Arber

## Abstract

Swiss microbial geneticist, Werner Arber shared the 1978 Nobel Prize in Physiology or Medicine with Hamilton Smith and Daniel Nathans for their discovery of restriction endonucleases.

Werner Arber was born in Granichen, Switzerland in 1929. Following a public school education, he entered the Swiss Polytechnical School in Zurich in 1949, working toward a diploma in natural sciences. There, his first research experience involved isolating and characterizing an isomer of chlorine.  Following graduation in 1953,  Arber joined a graduate program at the University of Geneva, taking on an assistanceship in electron microscopy (EM), in which he studied gene transfer in the bacterial virus (bacteriophage) lambda. Eventually encountering limitations with EM as a tool, he began using microbial genetics as a methodology for his studies. The study of microbial genetics had been possible for a relatively short time: DNA had been discovered to carry genetic information only a decade before he d entered the field.

After earning his Ph.D. in 1958, Arber continued to develop skills in microbial genetics, working with colleagues in the United States for a short time before returning to Geneva at beginning of 1960. There, he continued working on lambda transduction in *E. coli*, but found that the virus would not efficiently propagate. Recalling research done seven years earlier by Joe Bertani and Jean Weigle on "host-controlled restriction-modification", he realized there must be a host-controlled modification of the invading DNA, and sought to identify the mechanism.

Based on Grete Kallengerger s work that demonstrated degradation of both irradiated and non-irradiated phage lambda following injection in a host, Arber and his graduate student, Daisy Dussoix further investigated the fate of DNA, and found that restriction and modification (later determined to be postreplicative nuclotide methylation) directly affected DNA, but did not cause mutations.  They also found that theses were properties of the bacterial strains, and that both viral and cellular DNA were degraded.

Together, Arber and Dussoix reported their findings to scientific community in 1961 at the First International Biophysics Congress in Stockholm. Aber also presented the research to the Science Faculty of University of Geneva in 1962, earning the Plantamour-Prevost prize.  Based on his work and the work of others, he hypothesized that an enzyme in the host bacterium cut DNA into smaller pieces at specific sites, and methylase modified the host DNA to protect it from the digestive enzyme.  These theories were later confirmed by Urs Kuhnlein, who found that mutation of specific sites rendered the phage resistant to cleavage; Hamilton smith, who identified Type II endonuclease HindII; and Daniel Nathans, who used HindII to break the SV40 virus into 11 fragments, allowing him to determine its method of replication.

Since the discovery of restriction endonucleases, researchers have used them as tools to study the functions of genes of all types of organisms.  Restriction enzymes have also facilitated the study of gene functions and enabled production of substances of medical and nutritional importance.

Arber feels that in the next few decades we will learn much from the study of epigentics --factors that can affect the phenotype of an organism without changing the genetic information--.  He is proud that, in that studying restriction degradation and DNA methylation in the 1960s, he was among the first in studying epigenetic phenomenon.

**Figure:**